# Microbially facilitated nitrogen cycling in tropical corals

**DOI:** 10.1038/s41396-021-01038-1

**Published:** 2021-07-05

**Authors:** Thomas D. Glaze, Dirk V. Erler, Henri. M. P. Siljanen

**Affiliations:** 1 Centre for Coastal Biogeochemistry Research, School of Environment Science and Engineering, Southern Cross University, Lismore, NSW Australia; 2 Department of Environmental and Biological Sciences, University of Eastern Finland, Kuopio, Finland; 3 Department of Ecogenomics and Systems Biology, University of Vienna, Vienna, Austria

**Keywords:** Stable isotope analysis, Biogeochemistry, Biogeochemistry, Biogeochemistry, Microbial ecology

## Abstract

Tropical scleractinian corals support a diverse assemblage of microbial symbionts. This ‘microbiome’ possesses the requisite functional diversity to conduct a range of nitrogen (N) transformations including denitrification, nitrification, nitrogen fixation and dissimilatory nitrate reduction to ammonium (DNRA). Very little direct evidence has been presented to date verifying that these processes are active within tropical corals. Here we use a combination of stable isotope techniques, nutrient uptake calculations and captured metagenomics to quantify rates of nitrogen cycling processes in a selection of tropical scleractinian corals. Denitrification activity was detected in all species, albeit with very low rates, signifying limited importance in holobiont N removal. Relatively greater nitrogen fixation activity confirms that corals are net N importers to reef systems. Low net nitrification activity suggests limited N regeneration capacity; however substantial gross nitrification activity may be concealed through nitrate consumption. Based on *nrfA* gene abundance and measured inorganic N fluxes, we calculated significant DNRA activity in the studied corals, which has important implications for coral reef N cycling and warrants more targeted investigation. Through the quantification and characterisation of all relevant N-cycling processes, this study provides clarity on the subject of tropical coral-associated biogeochemical N-cycling.

## Introduction

Scleractinian corals harbour a broad range of bacteria, archaea, fungi, viruses and protists [1–5], which participate in a variety of interspecies relationships, ranging from mutualism to parasitism [6–9]. In addition to the well-studied symbiosis with *Symbiodiniaceae*, microbial associates service the ‘holobiont’ in a variety of capacities, including biogeochemical cycling [10–12]. The current weight of evidence suggests that microbes associated with the biogeochemical cycling of nitrogen (N) are potentially key members of tropical coral microbiomes [13, 14], though their function and impact on the coral holobiont remains ambiguous.

Coral reef systems are characterised by high primary productivity and low nutrient availability [15]. To survive chronic oligotrophy, corals rely on rapid assimilation and retention, particularly of nitrogen (N), which is the major limiting element for primary productivity in the ocean [16, 17]. Microbial associates with the capacity to assimilate and conserve N may play a major role in alleviating nutrient limitation [18, 19]. Conversely, microbes engaging in N release through denitrifying processes may strengthen host tolerance to nutrient replete conditions, such as those experienced during seasonal flood events [13], or help maintain favourable N to P ratio [20]. Furthermore, as the coral–*Symbiodiniaceae* association is largely maintained through provision and limitation of N from host to symbiont [15, 21, 22], N-cycling microbial associates may have the capability to reinforce and/or destabilise this relationship [13, 23].

The phylogenetic taxa and corresponding functional marker genes associated with N_2_ fixation, nitrification, denitrification, anammox and dissimilatory nitrate reduction to ammonia (DNRA) have all been reported within coral microbiomes [1–3, 24, 25]. With the exception of N_2_ fixation, our current understanding of coral-associated N-cycling is predominantly reliant on genetic and genomics studies, with little quantification of biogeochemical fluxes presented thus far. Few studies to date have measured nitrification and denitrification rates [19, 20], and rates of DNRA and anammox remain unquantified. While N-cycling processes have been quantified in cold-water corals [24], these organisms are physiologically and geographically distinct from tropical reef corals, and so their respective rates are unlikely to be analogous.

The coral organism is perhaps an unexpected setting to accommodate a vibrant N-cycling community, as many microbe-mediated N-cycling processes require anaerobic conditions. However, an assortment of microhabitats with disparate characteristics co-exist within the coral holobiont, providing an array of potentially suitable environments [26]. There is considerable spatial and temporal variation in O_2_, pH, nutrient concentration and light availability within and between coral microhabitats [27–30], which is often reflected in their distinct microbial assemblages [26].

While the significance of N_2_ fixation is clear [31, 32], it is unknown whether other N-cycling microbes play an active role in controlling N availability, or if they simply reflect opportunistic responses to favourable nutrient perturbations. Here we aimed to detect and quantify rates of denitrification, nitrification and DNRA from coral microbiomes on a tropical island system in the Great Barrier Reef. Measured rates were compared with other coral N-cycling processes including assimilation of fixed N_2_ hereby referred to as diazotroph-derived nitrogen (DDN), NH_4_^+^ assimilation and organic/inorganic N fluxes.

## Materials and methods

### Study site and collection

This study was conducted at One Tree Island and the associated reef lagoon in the southern section of the Great Barrier Reef (23° 30.39′ S 152° 05.48′ E) during November 2017. One Tree Island Reef is an emergent platform characterised by lagoonal patchwork reefs and a prominent reef crest, which isolates the lagoon for roughly 5 h each tide [33], elevating NH_4_^+^ and NO_3_^−^ concentrations above surrounding oceanic water [34]. Nutrient concentrations were measured at 0.23 ± 0.20-μmol L^−1^ NH_4_^+^, 0.27 ± 0.17-μmol L^−1^ NO_3_^−^, temperature diel variation ranged from 23 to 27 °C and dissolved oxygen (DO) from 125 to 405 μmol L^−1^. Here we conducted multiple ^15^N tracer incubation experiments on several coral species. Terminal fragments (4–6 cm) were collected at 1–3-m depth from a single representative colony of six scleractinian coral species; *Acropora grandis*, *Acropora pulchra*, *Porites cylindrica, Montipora digitata, Isopora elizabethensis* and *Isopora cuneata*. Additional fragments were collected from each for DO flux, DNA extractions and surface area (SA) calculations. Detached fragments were pre-incubated for 24 h in 60-L outdoor flowthrough holding tanks, covered by 15% weave shade cloths to attenuate light intensity and prevent rapid DO fluxes. Holding tanks received 5-L min^−1^ water via 50,000-L lagoon fed header-tanks. Ammonium (2.07 ± 0.80 μmol L^−1^) and nitrate (2.22 ± 0.70 μmol L^−1^) concentrations in the header-tank water were markedly higher than measured lagoon samples. While these values are within the reported range of DIN for OTI lagoon [33], the considerable DIN elevation relative to in situ concentrations means that N-cycling process rates are recorded under moderately eutrophic conditions. The influence of such eutrophy on respective N-cycling process rates fluxes is discussed below.

### Experimental details

Following pre-incubation, coral fragments were divided into individual 250-mL glass jars containing unfiltered header-tank water. Jars were sealed without headspace via Teflon septa and inverted to allow maximal light penetration. Replicate fragments from the representative colony of each species were subjected to one of three enriched treatments: ^15^N_2_, ^15^NO_3_^−^ and ^15^NH_4_^+^; each in triplicate. Additional duplicate fragments were incubated without enriched tracers to provide the isotopic baseline and background variability for flux and assimilation calculations. Further unenriched fragment laden vials (in duplicate) were incubated and measured hourly with a Hach LDO probe to track DO production and consumption. This incubation framework was conducted under both natural light and dark conditions with separate fragment batches to evaluate diel variation across N-cycling rates. Incubation times were terminated once DO concentrations reached the limits of on-site variability, ranging from 3.75 to 5 h in light incubated corals, and 5–8.75 h in dark incubated corals. For the ^15^N-N_2_ enrichment, the ^15^N_2_ dissolution technique [35] was applied, where 10 mL of ^15^N-N_2_ stock solution was injected (with venting) to each 250-mL jar. Stock solution was prepared through addition of 25-mL pure ^15^N-N_2_ gas (>98% Cambridge Isotope Laboratories, lot no. I-19168A) to 500 mL 0.22-µm filtered and degassed (45 min) seawater. For the ^15^NO_3_^−^ and ^15^NH_4_^+^ treatments, incubation jars received 1-mL injections of >98% ^15^N-KNO_3_ or ^15^N-NH_4_Cl stock solutions, totalling 3 μmol L^−1^ of additional DIN in each respective treatment.

### Sampling and analysis

Filtered water samples (0.22 µm) were collected from control treatment jars prior to and post incubation, allowing characterisation of nutrient perturbations over the experimental period. Concentrations of NO_*x*_ (NO_3_^−^ + NO_2_^−^), NH_4_^+^ and total dissolved N (TDN) were measured colorimetrically via a Lachat Quikchem 8500 Series 2 Flow Injection Analyser. As NO_2_^−^ is negligible in reef waters, NO_*x*_ is presumed to represent NO_3_^−^ only. Dissolved organic N (DON) was calculated as: DON = TDN − (NH_4_^+^ + NO_*x*_).

Denitrification was measured as the production of ^15^N-N_2_ in jars subjected to ^15^NO_3_^−^ or ^15^NH_4_^+^ addition. Following incubations, water samples were collected, added to duplicate 12-mL exetainers without headspace, and killed using saturated HgCl_2_ solution (20 µL ~ 8% w/v). Vials were headspaced with 2-mL He and left to equilibrate overnight. Exetainer headspace samples (10 µL) were analysed for ^15^N-N_2_ on a Thermo Trace GC Ultra with a 25 m × 0.32-mm PoraPLOT Q column interfaced to a Thermo Delta V Plus isotope ratio mass spectrometer (IRMS) (precision ± 0.15‰). Sample gases were passed over a heated copper reduction column prior to separation on the column, reducing all N_2_O and NO present to N_2_. Given the high *m*/*z*30 generated during ionization in the IRMS, the production of ^30^N_2_ could not be accurately determined, therefore the rate of total N_2_ production was calculated via the production of ^29^N_2_ only [36] after both ^15^N-NO_3_^−^ and ^15^N-NH_4_^+^ addition in the respective treatments. This approach assumes no contribution from anammox to N_2_ production. While we found no molecular evidence of anammox (see results), a previous study has detected *hzsA* and *hzo* genes within coral microbiomes [25]. The presence of anammox would overestimate total N_2_ production. Note that N_2_ production following ^15^N-NO_3_^−^ addition signifies heterotrophic denitrification, while N_2_ production following ^15^N-NH_4_^+^ represents some combination of coupled nitrification–denitrification, and potentially N_2_O production via nitrifier nitrification and nitrifier denitrification.

For nitrification rates, filtered water samples (0.22 µm) were collected from control and ^15^N-NH_4_^+^ treatment jars. Nitrification was determined via the production of ^15^N-NO_3_^−^ in the ^15^N-NH_4_^+^ treatment [24]. The N isotope signature of NO_3_^−^ (*δ*^15^N-NO_3_^−^) was determined via the denitrifier technique [37], which converts NO_3_^−^ to N_2_O. The N isotope signature *δ*^15^N ((*δ*^15^N (in permil, ‰) = 1000*((^15^N/^14^N)_sample_/(^15^N/^14^N)_reference_ − 1), where the ^15^N/^14^N reference is N_2_ in air). Liberated N_2_O was purified in liquid N_2_ with a custom-built purge and trap system and the *δ*^15^N-N_2_O was measured via a Thermo Delta V Plus IRMS coupled to a Thermo Fisher GasBench II. The *δ*^15^N-NO_3_^−^ values were standardised against IAEA-NO-3 (*δ*^15^N-NO_3_^−^ of 4.7‰) with an analytical precision of 0.2‰. Nitrification values represent the appearance of ^15^N-NO_3_^−^, signifying net nitrification, as NO_3_^−^ subsequently assimilated or immobilized remains undetected.

Coral fragments were rinsed with header-tank water, shaken dry, weighed and frozen at −20 °C. To determine the respective assimilation rates of DDN and NH_4_^+^ assimilation, fragments were ground to a powder with a stainless-steel mortar and pestle, freeze dried and weighed (100 mg) into tin capsules for bulk *δ*^15^N analysis. This was performed via elemental analysis on a Thermo Finnigan Flash EA 1112 coupled to a Thermo Delta V Plus IRMS via a Thermo Conflo III, providing *δ*^15^N (precision ± 0.15‰) and %N (precision ± 1% coefficient of variation). Respective rates of DDN and NH_4_^+^ assimilation were determined using the difference in ^15^N content between control coral fragments and those exposed to ^15^N_2_ or ^15^NH_4_^+^. Volumetric rate of DDN and NH_4_^+^ assimilation (*ρ*) was presented as µmol m^−2^ d^−1^, calculated using:

1 $$\rho = \left[ {\frac{{A_S - A_C}}{{(A_i - A_C)t_{{{{\mathrm{inc}}}}}S}}} \right] \times \overline {PN} \times 24 \times 10^4$$

where *A*_*s*_ = ^15^N Atom% enriched fragments, *A*_*c*_ = ^15^N Atom% of natural abundance control fragments, *A*_*i*_ = ^15^N Atom% initial label concentration of either ^15^NH_4_^+^ or ^15^N_2_, *t*_inc_ = incubation time (h), $\overline {PN}$ = µmol particulate N per fragment (assuming no change) and *S* = fragment SA (cm^2^). Atom% (*A*) and particulate nitrogen (*PN*) calculations were derived from Montoya [38].

2 $$A = 100 \times \left[ {\frac{{(10^{ - 3}\delta ^{{{{\mathrm{15}}}}}N + 1)({\,}^{15}N/{\,}^{14}N)_{{{{\mathrm{atmosphere}}}}}}}{{1 + (10^{ - 3}\delta {\,}^{15}N + 1)({\,}^{15}N/{\,}^{14}N)_{{{{\mathrm{atmosphere}}}}}}}} \right]\,{{{\mathrm{and}}}}\,\overline {PN} = \left[ {\frac{{W \times N}}{{100}}} \right] \times \left[ {\frac{{10^6}}{{14}}} \right]$$

where *W* is the fragment weight (g) and *N* is the fragment %N. Nitrogen fixed and subsequently released as DIN, DON or suspended particles was not evaluated.

DNRA process rates were not measured directly, instead we evaluated potential DNRA using an NO_3_^−^ mass balance model for both light and dark scenarios, according to the following:

3 $${{{{{{\rm{NO}}}}_3}^{-}{{{\rm{assimilation}}}}}} = {{{{{{\rm{NO}}}}_3}^{-} {{{\rm{net}}}}\;{{{\rm{flux}}}}}} + {{{{{\rm{Gross}}}}\;{{{\rm{nitrification}}}}{\mbox{-}}{{{\rm{DNRA}}}}}}$$

And therefore:

4 $${{{\mathrm{DNRA}}}} = {{{{\rm{NO}}}}_3}^{-} {{{\rm{net}}}}\;{{{\rm{flux}}}} + {{{\rm{Gross}}}}\;{{{\rm{nitrification}}}} - {{{{\rm{NO}}}}_3}^{-} {{{\rm{assimilation}}}}$$

This formula accounts for all possible pathways of NO_3_^−^ flux. Nitrate net flux is calculated using the change in NO_3_^−^ concentration over the incubation period. Gross nitrate assimilation was not measured directly but can be estimated using measured NH_4_^+^ assimilation data (via ^15^NH_4_^+^ amendment) and the ratio of NH_4_^+^ to NO_3_^−^ assimilation (Eq. (6)).

5 $${{{\rm{DNRA}}}} = {{{{\rm{NO}}}}_3}^{ -}\; {{{\rm{net}}}}\;{{{\rm{flux}}}} + {{{\rm{Gross}}}}\;{{{\rm{nitrification}}}}-\left({{{{\rm{NH}}}}_4}^{ +}\;{{{\rm{assimilation}}}}/R \right)$$

where *R* is the NH_4_^+^:NO_3_^−^ assimilation ratio. *R* can be expressed as %DIN, representing NH_4_^+^ assimilation as a percentage of total DIN assimilation

6 $$\% {{{\rm{DIN}}}} = {{{{\rm{NH}}}}_4}^{ +}\, {{{\rm{assim.}}}}/\left({{{{\rm{NH}}}}_4}^ {+}\,{{{\rm{assim.}}}} + \left({{{{\rm{NH}}}}_4}^{ +}\,{{{\rm{assim.}}}}/R\right)\right) \times 100$$

Gross nitrification was represented as a percentage of NH_4_^+^ assimilation. A matrix of potential DNRA rates was then calculated and constrained using literature values of gross nitrification [19] and conservative estimates of NO_3_^−^ assimilation [39, 40].

All N-cycling rates were normalised to coral SA with units of µmol N m^−2^ d^−1^. We determined SA through 3D-scanning of additional fragments from each tested colony (*n* = 6) using a David SLS-3 laser scanner, quantified using Autodesk Netfabb Premium meshing software. Average SA/weight ratios from these representatives were utilized as species-specific SA/weight ratios and applied to weighed experimental fragments.

### Captured metagenomics

We used *P. cylindrica* as a model species to characterise both the abundance and species composition of nitrogen cycling functional marker genes related to nitrogen fixation, nitrification, denitrification, anammox and DNRA through the use of a custom designed captured metagenomics tool. Two *P. cylindrica* fragments were collected and immediately frozen (−20 °C), ground and homogenized in liquid N_2_ with a stainless-steel mortar and pestle. DNA extractions were conducted on the resultant slurries using Qiagen DNeasy Powerbioflim minikit. DNA concentration and purity were quantified using a Qubit fluorometer and Nanodrop 2000c spectrophotometer, respectively. We utilized the NimbleGen SeqCap EZ protocol by Roche NimbleGen inc. The probe selection and validation are described in Supplementary methods, and bioinformatic data analysis was conducted as outlined in Aalto [41]. The relative frequency and taxonomic breakdown of captured gene hits are outlined in Supplementary table. The metagenomic data are deposited to the SRA database under the BioProject link PRJNA685986.

### Data analysis

Statistical analysis was conducted using Primer 7 software with the PERMANOVA+ add on. We conducted two factor PERMANOVAs based on Euclidean distance on a range of parameters (N_2_ production, net nitrification, DDN assimilation, NH_4_^+^ assimilation and net fluxes of NH_4_^+^, NO_3_^−^ and DON) to determine the influence of light regime and coral species. To conform datasets to normality log10 transformations were conducted. Type III (partial) sum of squares with permutation of residuals was used under a reduced model (999 permutations). Pairwise comparisons were conducted to determine the interaction between independent variables.

## Results

### Evidence for active denitrification and nitrification in tropical coral microbiomes

**Fig. 1:**
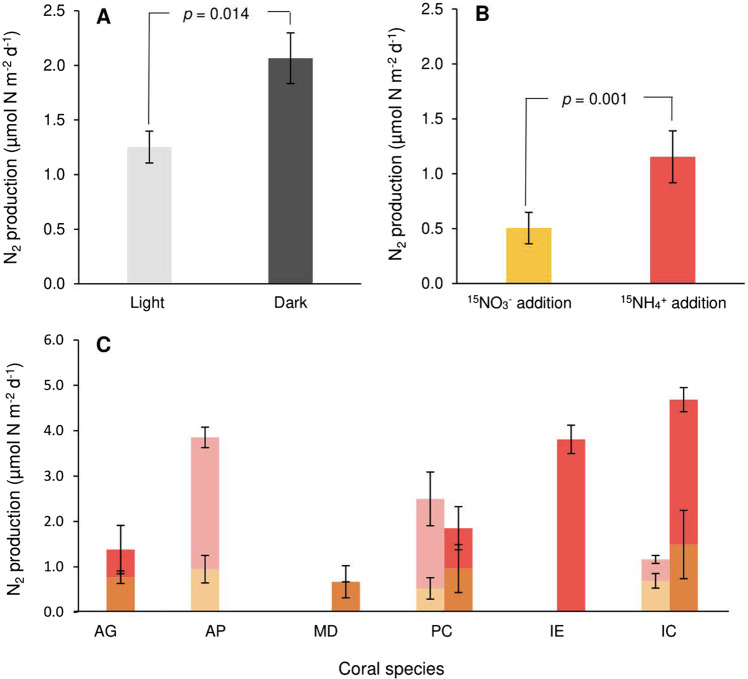
Total N_2_ production (µmol N m^−2^ coral surface area d^−1^) in ^15^NO_3_^−^ and ^15^NH_4_^+^ treatments. **A** Interspecies mean N_2_ production across light and dark incubations (accumulated over treatments). **B** Interspecies mean N_2_ production following ^15^NO_3_^−^ and ^15^NH_4_^+^ treatments averaged over light/dark incubations. **C** N_2_ production by coral species. Orange/yellow hues represent ^15^NO_3_^−^ addition, red/pink hues represent ^15^NH_4_^+^ addition. Light hues denote light incubated corals. Dark hues denote dark incubated corals. All error bars as standard error.

Denitrification, defined as the cumulative release of N_2_, N_2_O and NO, was recorded in all tropical coral species tested, *x̄* = 1.56 µmol N m^−2^ d^−1^. Corals incubated under dark conditions registered greater N_2_ production (*x̄* = 2.07 µmol N m^−2^ d^−1^), than those under light conditions (*x̄* = 1.25 µmol N m^−2^ d^−1^, *p* = 0.014) (Fig. 1A).

N_2_ produced following ^15^NO_3_^−^ addition (0.50 µmol N m^−2^ d^−1^) was considerably lower than from ^15^NH_4_^+^ addition (1.15 µmol N m^−2^ d^−1^, *p* = 0.001) (Fig. 1B), though this varied greatly within and between tested colonies (Fig. 1C). Total N_2_ production was negligible in comparison with other holobiont N release mechanisms such as estimated NH_4_^+^ release (*x̄* = 1993.4 µmol N m^−2^ d^−1^) and net DON flux (*x̄* = 448.5 µmol N m^−2^ d^−1^), representing just 0.07% TDN release.

**Fig. 2:**
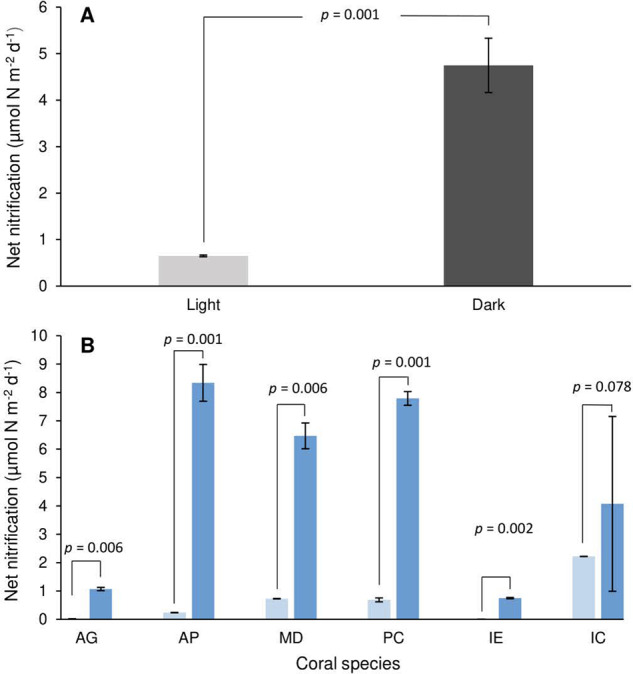
Net nitrification rate measurements. **A** Mean interspecies net nitrification in light and dark incubations. **B** Net nitrification by species. Light hues represent light incubated corals. Dark hues represent dark incubated corals. Error bars as standard error.

Net nitrification, identified as the appearance of ^15^N-NO_3_^−^ in ^15^N-NH_4_^+^ labelled incubations, was observed in all tested colonies, *x̄* = 2.70 µmol N m^−2^ d^−1^. Significantly higher rates were recorded under dark (*x̄* = 4.75 µmol N m^−2^ d^−1^) than light incubated corals (*x̄* = 0.65 µmol N m^−2^ d^−1^, *p* < 0.001) (Fig. 2A), which was observed in all species but *I. cuneata* (*p* = 0.078) (Fig. 2B). While all species presented measurable net nitrification rates under both light and dark conditions, significant interspecies differences were observed (*p* < 0.009). Despite modest net nitrification rates, the highly enriched *δ*^15^N-NO_3_^−^ of the remaining NO_3_^−^ pool indicates considerably greater gross nitrification activity. The *δ*^15^N-NO_3_^−^ shows elevated enrichment from dark (*x̄* = 325.97‰) than light incubations (*x̄* = 40.55‰), despite the longer incubation times depleting the nitrate concentration. This suggests that dark upregulated net nitrification accurately reflects diel patterns in gross nitrification, and is not simply an artefact of disparate NO_3_^−^ assimilation rates. In the absence of gross nitrification measurements, we accept the rates of corals incubated at One Tree Island may be within the range reported by Wafar et al. (1990) [19]. For dark incubations, at ≈17% of NH_4_^+^ assimilation, this would equal *x̄* = 196.6 µmol N m^−2^ d^−1^. Assuming *δ*^15^N-NO_3_^−^ enrichment scales linearly with gross nitrification between light and dark incubations, daytime gross nitrification can then be estimated via

7 $${{{\mathrm{GNIT}}}}_{{{{\mathrm{light}}}}} = {{{\mathrm{GNIT}}}}_{{{{\mathrm{dark}}}}}/\left( {\frac{{\delta ^{15}{{{{\mathrm{NNO}}}}_3}^ {-}\, dark}}{{\delta ^{15}{{{{\mathrm{NNO}}}}_3}^ {-}\, light}}} \right)$$

where GNIT_light_ = light gross nitrification, GNIT_dark_ = dark gross nitrification, *δ*^15^N-NO_3_^−^ dark = *δ*^15^N-NO_3_^−^ in dark incubations and *δ*^15^N-NO_3_^−^ light = *δ*^15^N-NO_3_^−^ in light incubations. This delivers a rough gross nitrification estimation of 24.5 µmol N m^−2^ d^−1^.

### Other N-cycling pathways

**Fig. 3:**
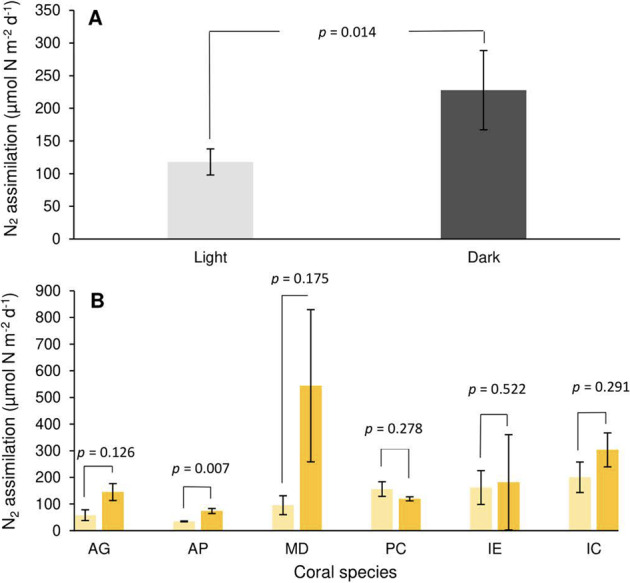
Diazotroph-derived nitrogen (DDN) assimilation rates. **A** Mean interspecies DDN assimilation during light and dark scenarios. **B** DDN assimilation across coral species. Light hues represent light incubated corals. Darker hues represent dark incubated corals. Error bars as standard error.

Assimilation of N via N_2_ fixation (DDN) occurred in all tested colonies, with an interspecies mean of 172.8 µmol N m^−2^ d^−1^. Across species, assimilation of DDN was significantly greater in dark (*x̄* = 227.8 µmol N m^−2^ d^−1^) than light incubated corals (*x̄* = 117.9 µmol N m^−2^ d^−1^, *p* = 0.014) (Fig. 3A); however, within species, this tendency was only significant in *A. pulchra* (*p* = 0.007) (Fig. 3B). We find significant interspecies variability in DDN assimilation (*p* = 0.012).

N_2_ fixation represents a non-negligible N source to the coral N budget, where DDN equates to 7.6% of the quantity N-NH_4_^+^ assimilated. The relative contribution of DDN, when compared with NH_4_^+^ assimilation as a nutritional component was much greater under dark (19.7%) than light incubations (3.46%), and mostly consistent across species (10.5 ± 0.7%) with the exception of *A. pulchra* (1.4%). Nitrogen gained through DDN assimilation outpaced nitrogen lost through N_2_ production by a factor of 104:1, signifying corals are net importers of N to reef systems. This was consistent across both light (94:1) and dark conditions (110:1) but variable between species, ranging from *A. pulchra* (28:1) to *M. digitata* (954:1). Note that this study did not measure gross N_2_ fixation, as fixed ^15^N residues released in the form of DIN, DON and suspended particles were not evaluated. This ratio of DDN assimilation:N_2_ release therefore likely underestimates the total net import of N to reef systems from coral organisms.

**Fig. 4:**
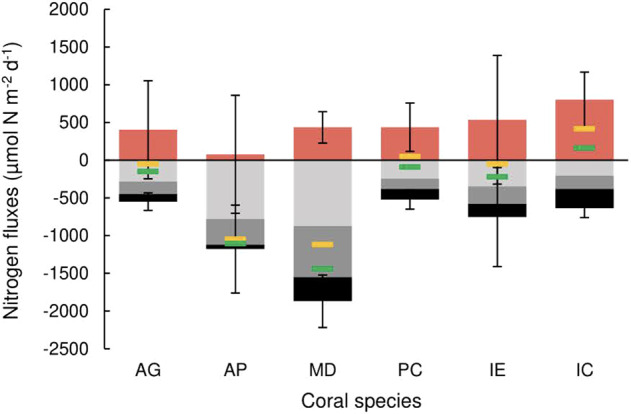
Net fluxes of DON (red), NO_3_^−^ (light grey), NH_4_^+^ (dark grey) and N_2_ (black) within the coral holobiont. Positive values denote net positive flux (release), negative values denote net negative flux (uptake). Yellow markers denote dissolved N flux excluding DDN, green markers denote dissolve N flux including DDN. Error bars as standard error.

All measured coral colonies assimilated NH_4_^+^ into biomass, *x̄* = 2283.5 µmol N m^−2^ d^−1^, with more rapid assimilation occurring under light (*x̄* = 3410.2 µmol N m^−2^ d^−1^) than dark incubations (*x̄* = 1156.3 µmol N m^−2^ d^−1^, *p* < 0.001), and significant differences between species (*p* < 0.001). Net uptake of NH_4_^+^ was recorded across all species (*x̄* = 289.8 µmol N m^−2^ d^−1^), where apparent differences between light (*x̄* = 392.5 µmol N m^−2^ d^−1^) and dark incubations (*x̄* = 187.2 µmol N m^−2^ d^−1^) were statistically insignificant (*p* = 0.084). The net flux of NH_4_^+^ represents only 7–20% of measured NH_4_^+^ assimilation values across species, signifying considerable NH_4_^+^ release and assimilation occur concurrently. Assuming this is not a methodological artefact, combined NH_4_^+^ release mechanisms (NH_4_^+^ waste/mineralisation/DNRA) must account for the remaining 80–90+%. Net uptake of NO_3_^−^ was also recorded across all species (*x̄* = 453.3 µmol N m^−2^ d^−1^) with greater uptake occurring in dark (*x̄* = 591.1 µmol N m^−2^ d^−1^) over light incubations (*x̄* = 316.7 µmol N m^−2^ d^−1^ *p* = 0.001). All species exhibited net DON release, (*x̄* = 448.5 µmol N m^−2^ d^−1^) with significant variation within and between tested colonies yet no discernible differences between light (*x̄* = 426.2 µmol N m d^−1^) and dark conditions (*x̄* = 448.5 µmol N m^−2^ d^−1^, *p* = 0.668). The net flux of N_2_, calculated as: N_2_ net flux = DDN assimilation − N_2_ production accounts for a considerable proportion of the coral dissolved N budget, equivalent to 23% of net DIN flux (range = 4.7–65%) and can modify the dissolved N budget status from net release to net uptake (Fig. 4).

### DNRA activity

**Fig. 5:**
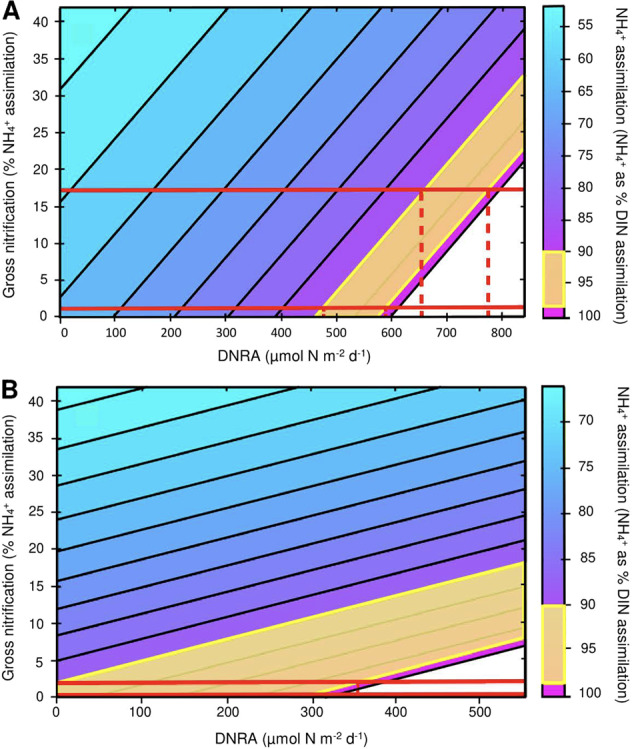
NO_3_^−^ mass balance models depicting potential DNRA activity. These models estimate DNRA (*x*-axis) over a range of gross nitrification (*y*-axis) and NH_4_^+^:NO_3_^−^ assimilation ratios (secondary axis). The ratio of NH_4_^+^:NO_3_^−^ assimilation is represented using NH_4_^+^ as % assimilation. Yellow bars highlight the probable range of %DIN [39, 40]. Red solid lines represent the range of likely gross nitrification estimates [19]. Red dashed lines illustrate estimated DNRA under various %DIN and gross nitrification scenarios. **A** DNRA activity under dark conditions. **B** DNRA activity under light conditions.

The estimation of DNRA activity is largely influenced by the ratio of NH_4_^+^ assimilation to NO_3_^−^ assimilation. To date, the only studies to have tracked gross uptake of both NH_4_^+^ and NO_3_^−^ within one coral species show that NH_4_^+^ assimilation is 1–2 orders of magnitude higher than NO_3_^−^ [39, 40], meaning NH_4_^+^ accounts for 90–99% of total DIN assimilation. Assuming this tendency applies to the current study, and using net nitrification values recorded herein (<1% NH_4_^+^ assimilation) as inputs for gross nitrification in the NO_3_^−^ mass balance model, DNRA activity equates to ~460–600 µmol N m^−2^ d^−1^ in dark incubations (Fig. 5A). Actual gross nitrification rates are likely to be much higher, as net nitrification rates do not account for assimilation of nitrified NO_3_^−^. Using the only quantified gross nitrification rates in the literature at ≈17% of NH_4_^+^ assimilation [19] as inputs instead, DNRA activity under dark conditions ranges from 660 to 780 µmol N m^−2^ d^−1^ (Fig. 5A). Under all available combinations of literature NH_4_^+^ to NO_3_^−^ assimilation ratios and nitrification rates, dark associated DNRA presents a potentially significant pathway of nutrient retention. Daytime DNRA is more difficult to estimate, although ostensibly less than under dark conditions. Measured net nitrification rates and *δ*^15^N-NO_3_^−^ values are much lower under light than dark conditions, and NH_4_^+^ assimilation is already an order of magnitude greater than NO_3_^−^ net flux. Using either our measured net nitrification values or previously outlined extrapolations of daytime gross nitrification (see: ‘Results’, nitrification) as gross nitrification inputs, estimated daytime DNRA remains in the range of 0–300 µmol N m^−2^ d^−1^ (Fig. 5B).

### Captured metagenomics

**Fig. 6:**
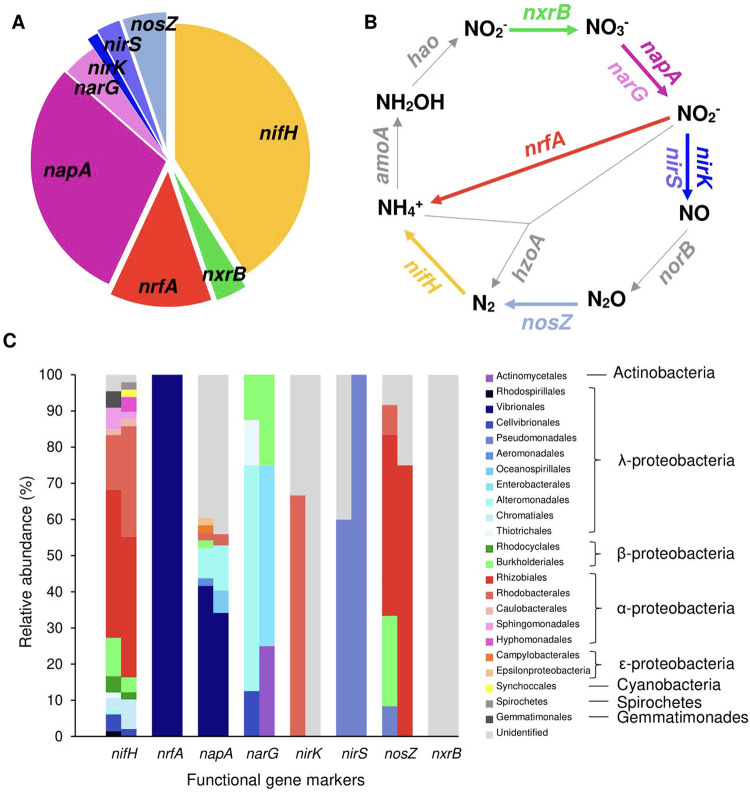
Captured metagenomics functional marker gene hits. **A** Specific N-cycling gene hits as proportion of total captured N-cycling genes. Functional marker gene encoding as follows: nitrogen fixation—yellow, nitrite oxidation (nitrification)—green, nitrite reduction (DNRA)—red, nitrate reduction (DNRA/denitrification)—purple, NO/N_2_O/N_2_ reduction (denitrification)—blue. **B** Process pathways for various N-cycling functional genes. Grey arrows represent genes not detected in *P.cylindrica*. **C** Class and order diversity of captured N-cycling genes hits, and relative abundance of functional marker genes. Both fragments are represented via split bars.

The results from the captured metagenomics analysis of *P. cylindrica* largely align with, and contextualise the biogeochemical evidence of coral N-cycling, yet also present additional lines of enquiry. The captured metagenomic analysis did not detect functional genes associated with anammox (*hzoA*) or ammonia oxidation (*amoA, hao*), yet found some nitrite oxidizing functional maker genes (*nxrB*), comprising 3.6% of N-cycling gene hits (Fig. 6A). A high proportion of captured hits were nitrate reduction genes, *napA* (29.4%), and *narG* (4.1%) (Fig. 6A). While these genes conduct the initial and rate-limiting step of both denitrification and DNRA, the major taxonomic groups identified here for nitrate reduction do not possess the requisite enzymatic machinery for denitrification. Microbes identified in captured *napA* hits were dominated by Vibrionales, which generally possess *nrfA* and not *nirS/nirK* [42] permitting NO_2_^−^ reduction to NH_3_, but not NO, thereby performing DNRA under O_2_ limitation (Fig. 6B). Microbes identified in captured *narG* hits exhibit diverse nitrate reduction pathways, including; nitrate respiration, DNRA, coupled nitrification–denitrification and canonical denitrification. Captured *nrfA* hits represent 12.1% total N-cycling genes, and were entirely dominated by Vibrionales, primarily of the genus *Vibrio*. The remaining denitrifying marker genes had low relative concentrations, with *nirS*, *nirK*, *norB* and *nosZ* comprising 2.8%, 1.3%, 0% and 5.2% of N-cycling gene hits, respectively (Fig. 6A). Each of these gene pathways were dominated by distinct clades (Pseudomonadales, Rhodobacterales and Rhizobiales) (Fig. 6C). Taken together, this suggests DNRA and not denitrification is likely the dominant dissimilatory nitrate reduction pathway within the microbiome of *P. cylindrica*. At 40.1% of total N-cycling gene hits, the relative abundance of *nifH* provides further evidence that this process contributes significantly to the coral N-cycle (Fig. 6A). Captured *nifH* hits contained considerable species, order and class diversity, albeit dominated by Rhizobiales and Rhodobacterales lineages (Fig. 6C). We recognise that the *P. cylindrica* hologenome may exhibit distinct structural and functional differences to the remaining species, which are all representatives of *Acroporidae*. It is that with this in mind, we advise caution in directly attributing biogeochemical fluxes to specific microbial taxa across all studied species.

## Discussion

Based on the prevalence of the associated microorganisms and relevant functional genes, tropical coral microbiomes are postulated to process N through a variety of microbially facilitated pathways [3, 25, 43, 44]. To date many of these pathways have not been adequately quantified, and as such their relevance at the organism and ecosystem scale is largely unknown [13]. Below we discuss the presence of key microbial N-cycling processes operating within the holobiont including denitrification, nitrification, N_2_ fixation and DNRA.

### Denitrification and nitrification

**Fig. 7:**
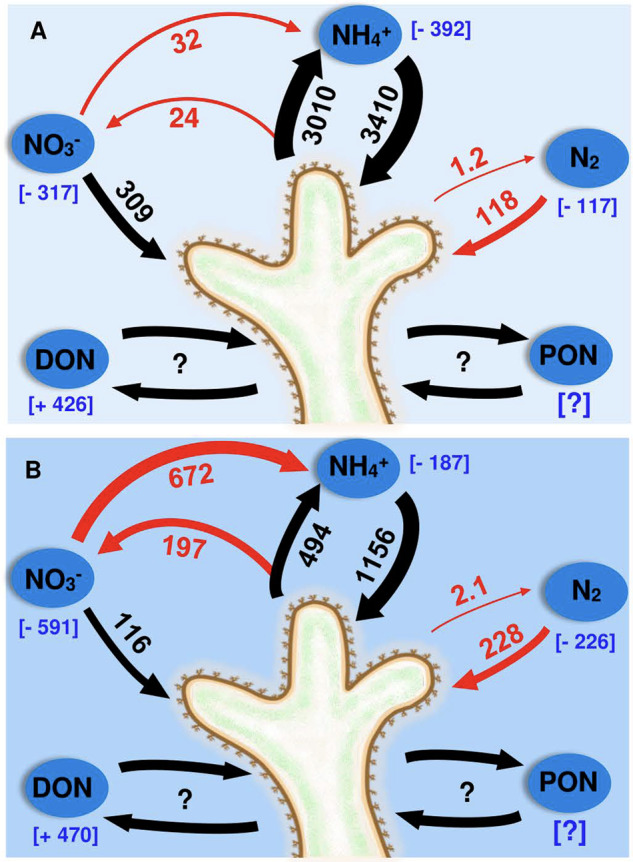
Compiled coral holobiont N-cycling processes and budget. Representative mass balance, displaying the magnitude of measured N-cycling pathways operating within live corals. Black arrows and values denote coral/*Symbiodiniaceae* pathways. Red arrows/values denote other microbially facilitated pathways. Blue values in square brackets denote net fluxes of respective substrates. All values are in µmol N m^−2^ d^−1^. **A** Coral N-cycling under light conditions. **B** Coral N-cycling under dark conditions.

Production of N_2_ was recorded in all species tested at One Tree Island. Denitrification rates were of comparable magnitude to those of previous tropical coral measurements [20, 45] and also those of the cold-water coral *Lophelia pertusa* [24], suggesting that denitrification is a ubiquitous feature of coral microbiomes, irrespective of physiology and habitat. Previous molecular studies confirm that denitrifying organisms reside in coral mucus [46], tissue [44] and speculatively within gastric cavities [28]. Denitrification activity was hypothesised to upregulate during the night, when depleted O_2_ boundary conditions provide favourable environmental settings for anaerobic processes [46, 47]. While our interspecies mean does show dark upregulation (Fig. 1A), this trend is not observed across all species (Fig. 1C). Similarly, while NH_4_^+^ is the preferred substrate for N_2_ production (Fig. 1B), this varies wildly (Fig. 1C) suggesting that the disparate physicochemical environments between coral species may favour divergent pathways. Multiple authors proposed that denitrification may present a meaningful pathway for N release from the coral holobiont under eutrophic stress [13, 46]. The rates presented here, and their relative contribution to TDN release (0.07%) infer N_2_ production is of limited functional importance to the coral holobiont. We would expect the relatively eutrophic conditions under which these incubations were performed to stimulate denitrification activity given the increased availability of substrate. Even these negligible values may therefore overestimate denitrification rates expected under typical OTI lagoon DIN concentrations. The limited importance of denitrification is reinforced by the captured metagenomics analysis on *P. cylindrica*, which indicates that the specific microbes possessing *napA*/*narG* are predominantly capable of DNRA, not denitrification. We also detected a relatively low proportion of *nirS*, *nirK*, *norB* and *nosZ* genes, with no organism identified as possessing a full suite of denitrifying genes (Fig. 6). Coral-associated denitrification is therefore likely an opportunistic response to the availability of inorganic N, rather than intrinsically important in the regulation of N availability (Fig. 7).

Nitrification activity was recorded in all species tested at One Tree Island. This aligns with the current literature, which suggest that coral nitrifying organisms may be ubiquitous, occurring in the mucus, tissue, skeleton and in interstitial spaces between branches [19, 46, 48]. Previous studies suggest that coral-associated nitrification activity would primarily be active during day light, when the DO saturation state of coral mucus is suitable for aerobic processes such as nitrification to occur [46]. Conversely, we find clear upregulation of dark nitrification activity across all species. This may be the result of the photoinhibitory effects of high light intensity breaching the light tolerance thresholds of the nitrifying organisms present [49]. Ammonia-oxidizing archaea (AOA) are especially susceptible to photoinhibition, providing further putative evidence that AOA are the dominant coral-associated ammonia oxidizers [43, 50].

Previous coral nitrification measurements outlined significant gross nitrification activity, equating to 17% NH_4_^+^ assimilation values under dark conditions [19]. The net nitrification values we present in this study are considerably lower, equating to 0.01% and 0.4% of NH_4_^+^ assimilation under light and dark scenarios, respectively. While these dissimilar outcomes may be the result of methodological differences, or regional/interspecies variability, findings may still be consistent between studies. The highly enriched *δ*^15^N-NO_3_^−^ of the nitrate pool following ^15^N-NH_4_^+^ incubation suggests that NO_3_^−^ consuming processes such as NO_3_^−^ assimilation, denitrification and DNRA may be masking high gross nitrification activity through consumption of nitrified NO_3_^−^. In addition, nutrient diffusion rates between the water column and endolithic nitrifiers may also obfuscate nitrification measurements. Nutrient diffusion rates similar to that of photoassimilates (24–48 h) may fall outside the incubation window, subsequently underestimating nitrification activity [51]. The true magnitude of gross nitrification in this study is therefore difficult to accurately determine, nevertheless it is clear that nitrified NO_3_^−^ is rapidly recycled.

Despite clear biogeochemical evidence of active nitrification, *amoA* and *hao* functional gene markers were not detected and could not be quantified in *P. cylindrica*. Given the high *δ*^15^N-NO_3_ enrichment level, and prior molecular evidence of coral-associated nitrification [43, 44, 46], we assume that *amoA* and *hao* are likely present, but remain undetected due to technical/methodological reasons, such as the specificity or sensitivity of the captured metagenomics probe.

### N_2_ fixation

Diazotrophs have previously been confirmed to reside in the coral mucus, tissue and skeleton microhabitats [52–56]. We find that N_2_ fixation is a ubiquitous feature of coral microbiomes, occurring in all coral species tested under both light and dark scenarios, which agrees with much of the previous research [1, 18, 57–59]. DDN has been repeatedly demonstrated as a significant N source for the coral host and *Symbiodiniaceae* [32, 53, 60]. Assimilation rates of DDN were substantial, and would potentially be greater still under standard OTI lagoon DIN concentrations, as elevated DIN can reduce or inhibit N_2_ fixation activity [61]. Relative to NH_4_^+^ assimilation, our reported N_2_ fixation rates may seem a modest contribution to TDN input; however, N_2_ net flux represents a significant share of the TDN net flux, suggesting that N_2_ fixation provides a disproportionate contribution to the coral N budget than DDN assimilation rates alone would imply (Fig. 4).

**Fig. 8:**
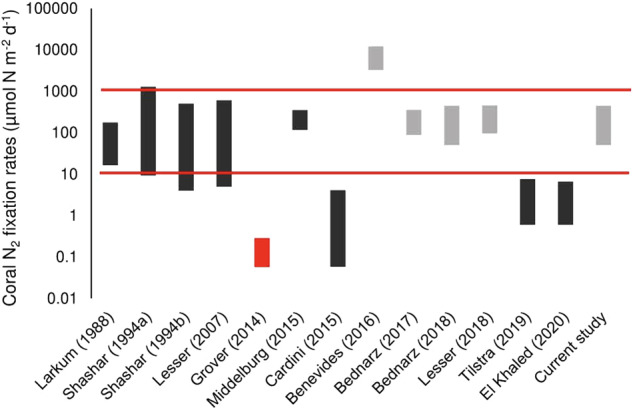
Approximate range of coral-associated N_2_ fixation values from recent articles, converted from a wide range of units to µmol N m^−2^ d^−1^. Dark grey bars represent studies, which used the acetylene reduction technique. Light grey bars represent studies, which used ^15^N_2_ tracer incubations. The study conducted by Grover et al. [59] has been filled red as it used the buddle dissolution technique for preparation of ^15^N_2_ stock solution, which considerably underestimates N_2_ fixation.

Our N_2_ fixation results compare favourably with most studies, which have utilized the ^15^N_2_ dissolution technique [31, 60, 62], and many which utilized acetylene reduction assays [52, 53, 63, 64] (Fig. 8). We find considerable overlap with Lesser et al. [31], which presents a similar range of N_2_ fixation values in the analogous high light, 5-m depth coral incubations (~220 µmol N m^−2^ d^−1^) as those presented here (54–320 µmol N m^−2^ d^−1^). Lesser found no influence of light regime on DDN assimilation rates, which aligns with five of the six coral species tested at One Tree Island. The community composition of identified diazotrophs also has parallels with previous studies, with abundant proteobacteria and little evidence of cyanobacteria [31, 57, 65]. With regard to net fluxes of bioavailable N, defined as the relative magnitudes of N_2_ fixing vs. N_2_ releasing processes, our study diverges with the available literature [20, 45]. Tilstra et al. [20] and El-Khaled et al. [45] report that the N_2_ net flux from coral holobionts is effectively nil, as N_2_ fixation and N_2_ production processes are balanced. While denitrification values are equivalent across studies, N_2_ fixation values reported in Tilstra and El-Khaled are ~2 orders of magnitude lower than most reported rates (Fig. 8). In light of this, we propose that N_2_ fixation provides an important N source to living corals, and determine through comparison with N_2_ production, coral holobionts are net N importers to coral reef systems under most scenarios (Fig. 7).

### DNRA

Elevated NO_3_^−^ availability in coral reefs [66, 67] coupled with more favourable uptake kinetics prompted earlier researchers to infer that NO_3_^−^ is the dominant DIN species incorporated into coral biomass [66]. However, studies tracing gross assimilation of NH_4_^+^ and NO_3_^−^ into coral tissue report that NH_4_^+^ is disproportionately assimilated at a rate 1–2 orders of magnitude above NO_3_^−^ [39, 40, 60]. In our light incubations, NH_4_^+^ assimilation is tenfold greater than net NO_3_^−^ uptake, consistent with Grover, implying gross NO_3_^−^ assimilation and net uptake may be synonymous. Under dark conditions, NH_4_^+^ assimilation was downregulated, while NO_3_^−^ net flux was simultaneously upregulated, providing a ratio of 2:1 and indicating gross NO_3_^−^ assimilation and net uptake are likely uncoupled. While it is possible that NO_3_^−^ assimilation upregulates under typical night time environmental conditions, this seems unlikely as coral NO_3_^−^ demand is driven by photosynthetic *Symbiodiniaceae* activity. A more parsimonious explanation is that another NO_3_^−^ consumption process is active at night, such as DNRA.

In the current study, DNRA activity provides a neat explanation for dark upregulation of net NO_3_^−^ uptake, and the absence of NO_3_^−^ uptake inhibition, the usual response to NH_4_^+^ availability [40, 68]. Records of ^15^N enrichment in the animal tissue fraction following ^15^NO_3_^−^ incubation experiments have perplexed authors, given the absence of the requisite nitrate reductase genes in coral hosts [40, 69]. We posit that ^15^NO_3_^−^ is converted into ^15^NH_4_^+^ through DNRA and subsequently assimilated into coral biomass in these cases. DNRA activity in coral holobionts is likely beneficial for coral fitness and function through reducing N limitation via the conversion of the more readily available DIN form (NO_3_^−^), to the more favourable and rapidly assimilable form (NH_4_^+^). The *Vibrio* taxa predominantly responsible for coral-associated DNRA may be not only physiologically relevant for the coral N budget, but also provide protection from thermal stress through altering the DIN composition [70], juxtaposing the disease and bleaching response associated with *Vibrio* lineages [71–73].

Multiple lines of evidence indicate potential DNRA activity. First, DNRA activity is substantiated by the relatively high *nrfA* and *napA* gene copy numbers measured here in *P. cylindrica*, and prior records of *nrfA* in *P. astreoides* [3]. The relatively high ratio of *nrfA* to *nosZ* we observed are also a key indicator of potential DNRA activity [42]. In addition, the environmental conditions present in corals at night provide suitable criteria to facilitate DNRA. Significant DNRA activity has already been demonstrated in coral reef sediments [74], and the conditions which stimulate DNRA and permit it to outcompete denitrification are numerous on living coral surfaces. DNRA activity favours hypoxic to anoxic boundary conditions, limited nitrate compared with organic carbon [75, 76], and high salinity and temperature [77, 78], all of which occur in coral tissue, mucus and skeletons at night [27–30, 79]. If DNRA activity is operating at the scale described in the mass balance model (460–780 µmol N m^−2^ d^−1^), this presents a significant N retention mechanism under dark conditions, functioning as the principal pathway of NO_3_^−^ depletion and contributing ~40–70% of the available NH_4_^+^ for assimilation (Fig. 7B).

## Conclusion

A diverse and dynamic N-cycling community is evidently a ubiquitous feature of the coral holobiont. Here we provide some of the first direct evidence that denitrification is active in living corals, although rates were low with respect to coral physiology and reef biogeochemistry (Fig. 7A, B). In addition, we present evidence of nitrification activity, positing recorded net nitrification rates likely underestimate gross nitrification activity. Importantly, we provide strong evidence that DNRA is an active N-cycling pathway in tropical corals, contributing a substantial component of assimilated NH_4_^+^ under dark conditions (Fig. 7B), and thereby operating at environmentally relevant scales. Finally, we corroborate the findings of earlier researchers, which highlight the importance of N_2_ fixation to coral N demand, and further identify corals as net N importers to reef systems (Fig. 7A, B). We advise readers exercise caution when applying the N-cycling rates presented herein to corals more broadly. We found high intraspecies and intracolony variability across many N-cycling processes, and markedly different patterns of activity between processes. Corals are heterogenous and dynamic systems, and further research is required to determine the relevant variables underpinning such variability.

## Supplementary information

Supplementary Methods

Supplementary Table
