# Supplementary material for: Microbially facilitated nitrogen cycling in tropical corals
Source: ISME J. 2021 Jul 5;16(1):68–77. doi: 10.1038/s41396-021-01038-1 (PMC8692614; doi:10.1038/s41396-021-01038-1)
Supplement: Supplementary file 1 — Supplementary Methods [file 41396_2021_1038_MOESM1_ESM.docx]

**Supplementary Methods:**

**Captured metagenomics of nitrogen cycling microbes**

Captured metagenomics used here on nitrogen cycling genes is based on the NimbleGen SeqCap EZ protocol (by Roche NimbleGen, Inc.). In brief, to develop a sequence capturing tool, all known sequences for nitrogen cycling genes involved in nitrogen fixation, nitrification, nitrate reduction, denitrification and DNRA were collected from publicly available (NCBI, WGS, Fungene; Alves et al. 2019; Graf et al., 2014) and private databases with gene specific probes into a local server (CSC, Espoo, Finland). These functional genes were then searched for selected areas of genes with nhmmer (Wheeler & Eddy, 2013) and tblastx (Altschul et al., 1990) with manually curated and aligned databases of known and isolated clades of each gene. These target databases (TDBs, in total about 900000 sequences) were then submitted into SeqCap pipeline after dereplication (Kushwaha, et al., 2015) with default parameters for designing up to six unique 50 mer probes for each dereplication (with 90% cut-off) of each target gene clusters. Information from these probes were then used to design NimbleGen SeqCap probe set (approximately 2M probes) for Targeted metagenomics. Performance and specificity of these probes for sequence capture was validated and tested against shot-gun and amplicon sequencing technology. It was shown that captured metagenomics showed much higher specificity and produced much higher diversity richness (Siljanen et al., 2020). For each sample libraries with Indexes and sequencing adapters were produced in CGR (Liverpool Centre for Genomic Research, Liverpool, UK). These libraries were then pooled together uniformly, quality controlled and hybridized to the NimbleGen SeqCap capturing probe set according manufacturer’s instructions at 47°C for 72 hours in CGR as previously described (Manoharan , et al., 2015). The sequencing produced about 2 million reads per sample. To diminish the computing effort the sequence files were split according to functional gene using the target gene hmmer profiles to search sequences of each functional gene maximum E-value cut-off (E < 0.001). The gene functions and closest cultured relatives of each sequence were searched against Swiss-prot database with tblastx algorithm in *diamond* sequence search accelerator (Buchfink et al., 2015). Annotation and identity information was further processed with *awk* in unix and in R to produce lists of community structures of each genes. For alpha diversity calculation, the data was normalized by subsampling to the smallest sequence count per gene.

**References:**

Altschul, S. F., Gish, W., Miller, W., Myers, E. W., & Lipman, D. J. (1990). Basic local alignment search tool. Journal of molecular biology, 215(3), 403-410. doi.org/10.1016/S0022-2836(05)80360-2

Alves, R.J.E., Minh, B.Q., Urich, T., von Haeseler, A., Schleper, C., 2018. Unifying the global phylogeny and environmental distribution of ammonia-oxidizing archaea based on amoA genes. Nature Communications 9, 1517. https://doi.org/10.1038/ s41467-018-03861-1.

Buchfink, B., Xie, C., & Huson, D. H. (2015). Fast and sensitive protein alignment using DIAMOND. Nature methods, 12(1), 59. doi.org/10.1038/nmeth.3176

Graf, D. R., Jones, C. M., & Hallin, S. (2014). Intergenomic comparisons highlight modularity of the denitrification pathway and underpin the importance of community structure for N_2_O emissions. PloS one, 9(12), e114118. doi.org/10.1371/journal.pone.0114118

Kushwaha, S. K., Manoharan, L., Meerupati, T., Hedlund, K., & Ahrén, D. (2015). MetCap: a bioinformatics probe design pipeline for large-scale targeted metagenomics. BMC bioinformatics, 16(1), 65. doi.org/10.1186/s12859-015-0843-2

Manoharan, L., Kushwaha, S. K., Hedlund, K., & Ahrén, D. (2015). Captured metagenomics: large-scale targeting of genes based on ‘sequence capture’ reveals functional diversity in soils. DNA Research, 22(6), 451-460. doi.org/10.1093/dnares/dsv026

Siljanen, H.M.P., Manoharan, L., Bagnoud, A., Hilts, A., Alves, R., Abby, S., Jones, C., Hallin, S., Ahren, D., Biasi, C., Schleper. C. 2020. Captured metagenomics on inorganic nitrogen and methane cycling reveals relative abundance and diversity of functional genes.

Wheeler, T. J., & Eddy, S. R. (2013). nhmmer: DNA homology search with profile HMMs. Bioinformatics, 29(19), 2487-2489. doi.org/10.1093/bioinformatics/btt403
