# Supplementary material for: Microbially facilitated nitrogen cycling in tropical corals
Source: ISME J. 2021 Jul 5;16(1):68–77. doi: 10.1038/s41396-021-01038-1 (PMC8692614; doi:10.1038/s41396-021-01038-1)
Supplement: Supplementary file 2 — Supplementary Table [file 41396_2021_1038_MOESM2_ESM.pdf]

		Frequency of marker gene hits													
		nifH		nrfA		napA		narG		nirK		nirS		nosZ	
**Order**	**Closest species**	**A**	**B**	**A**	**B**	**A**	**B**	**A**	**B**	**A**	**B**	**A**	**B**	**A**	**B**
Rhizobiales	*Mesorhizobium huakuii*	1													
Rhizobiales	*Martelella endophytica*	1													
Rhizobiales	*Bradyrhizobium japonicum*	1	1												
Rhizobiales	*Sinorhizobium meliloti*	1													
Rhizobiales	*Rhodopseudomonas palustris*	1	1												
Rhizobiales	*Hoeflea phototrophica*	17	6												
Rhizobiales	*Blastochloris viridis*	3													
Rhizobiales	*Fulvimarina pelagi*	2	5												
Rhizobiales	*Methylobacterium aquaticum*		1												
Rhizobiales	*M. rhodesianum*		3												
Rhizobiales	*Methylocella silvestris*		2												
Rhodobacterales	*Rhodovulum sulfidophilum*	2													
Rhodobacterales	*Roseibacterium elongatum*	4	2												
Rhodobacterales	*Hyphomonadaceae bacterium*	3													
Rhodobacterales	*Dinoroseobacter shibae*	1													
Rhodobacterales	*Tateyamaria omphalii*		4												
Rhodobacterales	*Loktanella vestfoldensis*		3												
Rhodobacterales	*Rhodobacter capsulatus*		1												
Rhodobacterales	*Planktomarina temperata*		5												
Rhodospirillales	*Asaia bogorensis*	1													
Caulobacterales	*Brevundimonas subvibrioides*	1	1												
Sphingomonadales	*Sphingomonas hengshuiensis*	2	1												
Sphingomonadales	*Porphyrobacter neustonensis*	2													
Hyphomonadales	*Hyphomonadaceae sp.*		2												
Rhodocyclales	*Dechloromonas aromatica*	1	1												
Rhodocyclales	*Methyloversatilis universalis*	2													
Burkholderiales	*Rhodoferax antarcticus*	7	2												
Alteromonadales	*Alteromonadaceae bacterium*	1													
Thiotrichales	*Beggiatoa leptomitiformis*	1													
Cellvibrionales	*Congregibacter litoralis*	3	1												
Chromatiales	*Thiodictyon syntrophicum*	1													
Chromatiales	*Thioflavicoccus mobilis*	1													
Chromatiales	*Marichromatium purpuratum*		4												
Synechococcales	*Cyanobium gracile*		1												
Gemmatimonales	*Gemmatimonas phototrophica*	3													
Spirochaetales	*Spirochaeta thermophil*		1												
Vibrionales	*Aliivibrio fischeri*			8	1	1	1								
Vibrionales	*Aliivibrio salmonicida*			2	0										
Vibrionales	*Vibrio parahaemolyticus*			14	2	8	4								
Vibrionales	*Vibrio vulnificus*			11	2	0	1								
Aeromonadales	*Aeromonas salmonicida*					1	0								
Campylobac.	*Campylobacter lari*					1	0								
Alteromonadales	*Colwellia psychrerythraea*					3	4								
Rhodobacterales	*Dinoroseobacter shibae*					1	1								
Burkholderiales	*Leptothrix cholodnii*					1	0								
Epsilonproteobac.	*Nitratiruptor sp.*					1	0								
Alteromonadales	*Saccharophagus degradans*					1	0								

Vibrionales	*Vibrio cholerae*					1	0								
Vibrionales	*Vibrio campbellii*					10	5								
Oceanospirillales	*Hahella chejuensis*					0	2								
Alteromonadales	*Marinobacter psychrophilus*							5							
Thiohalorhabdus	*Thiohalorhabdus denitrificans*							1							
Cellvibrionales	*Simiduia agarivorans*							1							
Enterobacterales	*Escherichia coli*								2						
Burkholderiales	*Burkholderia multivorans*							1							
Burkholderiales	*Ramlibacter tataouinensis*								1						
Corynebacteriales	*Nocardia seriolae*								1						
Rhodbacterales	*Rhodobacter sphaeroides*									2					
Pseudomonadales	*Pseudomonas aeruginosa*											1	2		
Pseudomonadales	*Pseudomonas stutzeri*											2	1		
Burkholderiales	*Achromobacter cycloclastes*													2	0
Rhizobiales	*Bradyrhizobium diazoefficiens*													3	3
Rhizobiales	*Brucella melitensis*													1	0
Rhodobacterales	*Paracoccus denitrificans*													1	0
Pseudomonadales	*Pseudomonas stutzeri*													1	0
Burkholderiales	*Ralstonia solanacearum*													1	0
Rhizobiales	*Rhizobium meliloti*													2	0
